# Artificial Intelligence in Primary Care: Support or Additional Burden on Physicians’ Healthcare Work?—A Qualitative Study

**DOI:** 10.3390/clinpract15080138

**Published:** 2025-07-25

**Authors:** Stefanie Mache, Monika Bernburg, Annika Würtenberger, David A. Groneberg

**Affiliations:** 1 Institute for Occupational and Maritime Medicine (ZfAM), University Medical Center Hamburg-Eppendorf (UKE), 20459 Hamburg, Germany; 2 Institute of Occupational Medicine, Social Medicine and Environmental Medicine, Goethe University Frankfurt, 60590 Frankfurt, Germany

**Keywords:** artificial intelligence, primary care, general practitioners, digital health, qualitative research, technostress, decision support, physician workload, doctor–patient relationship

## Abstract

**Background**: Artificial intelligence (AI) is being increasingly promoted as a means to enhance diagnostic accuracy, to streamline workflows, and to improve overall care quality in primary care. However, empirical evidence on how primary care physicians (PCPs) perceive, engage with, and emotionally respond to AI technologies in everyday clinical settings remains limited. Concerns persist regarding AI’s usability, transparency, and potential impact on professional identity, workload, and the physician–patient relationship. **Methods**: This qualitative study investigated the lived experiences and perceptions of 28 PCPs practicing in diverse outpatient settings across Germany. Participants were purposively sampled to ensure variation in age, practice characteristics, and digital proficiency. Data were collected through in-depth, semi-structured interviews, which were audio-recorded, transcribed verbatim, and subjected to rigorous thematic analysis employing Mayring’s qualitative content analysis framework. **Results**: Participants demonstrated a fundamentally ambivalent stance toward AI integration in primary care. Perceived advantages included enhanced diagnostic support, relief from administrative burdens, and facilitation of preventive care. Conversely, physicians reported concerns about workflow disruption due to excessive system prompts, lack of algorithmic transparency, increased cognitive and emotional strain, and perceived threats to clinical autonomy and accountability. The implications for the physician–patient relationship were seen as double-edged: while some believed AI could foster trust through transparent use, others feared depersonalization of care. Crucial prerequisites for successful implementation included transparent and explainable systems, structured training opportunities, clinician involvement in design processes, and seamless integration into clinical routines. **Conclusions**: Primary care physicians’ engagement with AI is marked by cautious optimism, shaped by both perceived utility and significant concerns. Effective and ethically sound implementation requires co-design approaches that embed clinical expertise, ensure algorithmic transparency, and align AI applications with the realities of primary care workflows. Moreover, foundational AI literacy should be incorporated into undergraduate health professional curricula to equip future clinicians with the competencies necessary for responsible and confident use. These strategies are essential to safeguard professional integrity, support clinician well-being, and maintain the humanistic core of primary care.

## 1. Introduction

Artificial intelligence (AI) is rapidly transforming healthcare systems worldwide, offering the potential to enhance diagnostic accuracy, optimize treatment plans, and improve patient outcomes [1,2]. In primary care, the frontline of healthcare delivery where physicians manage a broad spectrum of undifferentiated symptoms and complex chronic conditions, AI promises to support clinical decision-making, streamline administrative workflows, and strengthen preventive care [3,4]. Applications such as clinical decision support systems, risk prediction algorithms, and automated documentation tools are designed to improve diagnostic accuracy, increase efficiency, and reduce routine administrative burdens [2,5]. Initial evidence suggests that AI can enhance the identification of at-risk patients and facilitate evidence-based decisions [2,6].

Despite these promising prospects, the real-world integration of AI into primary care presents significant challenges. Physicians frequently report barriers including poor integration with existing workflows, limited usability, information overload, and lack of transparency or explainability in AI systems [7,8]. Concerns around data quality, algorithmic bias, and accountability in the event of diagnostic errors or AI-related harm also raise ethical and legal questions [9,10]. Beyond these technical and organizational issues, there is increasing recognition of the psychological impact of AI on clinicians. AI may contribute to “technostress”, the stress associated with digital complexity, interruptions, and role ambiguity, potentially exacerbating cognitive and emotional workload [11,12]. Furthermore, some physicians perceive AI as threatening their professional autonomy and the relational core of primary care, particularly when AI tools interfere with the doctor–patient relationship or shift decision-making away from the clinician [13,14].

Primary care physicians (PCPs) play a pivotal role as the first point of contact for most patients and as coordinators of comprehensive care [15]. Understanding PCPs’ perspectives on AI is therefore critical to ensure that technological innovations support, rather than burden, clinical practice. While quantitative studies have predominantly addressed the efficacy of AI applications, there remains a notable gap in qualitative research in exploring how PCPs experience and adapt to AI tools within their clinical workflows. This knowledge gap is especially urgent given the mounting pressures in primary care, including widespread clinician burnout, workforce shortages, and growing administrative demands [16,17]. For example, electronic health records (EHRs), while indispensable, contribute substantially to this burden by occupying a large portion of clinicians’ time [18,19].

Against this backdrop, AI emerges as a potentially valuable means to alleviate some of these pressures and enhance primary care delivery. Notably, although AI’s promise to improve healthcare quality and safety has been discussed for over two decades [20], the recent advent of generative AI technologies indicates that their integration into primary care will soon become more frequent and impactful in daily clinical practice [12]. However, to ensure sustainable and ethically responsible implementation, it is crucial to understand how PCPs perceive AI as either a supportive tool or an additional burden in their everyday work, and to identify the conditions that facilitate its successful integration.

Although AI applications in healthcare have been extensively studied for their technical performance and clinical outcomes, there is limited qualitative evidence regarding how PCPs experience and perceive the integration of AI into their everyday clinical workflows. Most existing research focuses on quantitative studies, while the psychological, professional, and practical impacts on PCPs remain underexplored. Understanding these perspectives is essential to address potential barriers, ethical concerns, and the risk of increased workload or stress, which could affect the acceptance and effective implementation of AI technologies in primary care.

### 1.1. Theoretical Background

Based on socio-technical systems theory and prior research on technostress, we hypothesized that the integration of AI into primary care entails not only technical change but also a fundamental transformation of workflows, decision-making, and professional roles. Understanding how physicians perceive and experience this transformation requires a theoretical framework that considers both the technological and the human dimensions of clinical work.

#### 1.1.1. Socio-Technical Systems Theory

This study is grounded in socio-technical systems theory, which posits that the successful implementation of technology in complex work environments, such as healthcare, requires alignment between social subsystems (e.g., users, practices, professional norms) and technical subsystems (e.g., tools, algorithms, digital systems) [21,22]. AI technologies are thus not neutral tools but integral components of a dynamic socio-technical system that continuously interacts with clinical routines, communication patterns, and institutional structures.

In this context, “misalignment” refers to situations where the design, implementation, or use of AI systems does not adequately fit or harmonize with the existing social and/or organizational context. This may manifest as workflow disruptions, increased cognitive or emotional burden for clinicians, compromised professional autonomy, or unintended shifts in roles and responsibilities [23]. Misalignment can undermine both the effectiveness of AI tools and user acceptance, ultimately impeding sustainable adoption.

The concept of misalignment remains under-theorized and insufficiently operationalized in current literature, particularly concerning AI integration in primary care. This gap justifies the use of qualitative research methods, as they enable an in-depth exploration of clinicians’ lived experiences and perceptions to uncover the nuanced dimensions of socio-technical misfits that quantitative measures or existing theories may overlook. Through inductive thematic analysis, this study seeks to identify and elaborate on these emergent concepts to inform both theory development and practical implementation strategies.

#### 1.1.2. Technostress and Digital Workload

The concept of technostress [24] helps explain how AI might contribute to psychological burden. Technostress refers to stress induced by the use or presence of technology, including feelings of overload, invasion, and complexity. In primary care, where physicians already face high time pressure and administrative demands, the introduction of AI systems, especially if poorly integrated, may exacerbate rather than relieve stress. Conversely, well-designed AI tools may reduce technostress by automating routine tasks and supporting decision-making.

#### 1.1.3. Technology Acceptance and Resistance

The Technology Acceptance Model (TAM) [25] and its later adaptations (e.g., UTAUT [26]) highlight perceived usefulness and ease of use as being critical for technology adoption. In healthcare, however, trust, accountability, and ethical concerns also significantly influence attitudes toward AI. Resistance may stem from lack of transparency; fear of professional de-skilling, or concerns about legal responsibility for AI-generated decisions.

By integrating socio-technical systems theory, technostress, technology acceptance models, and the sociology of professional identity, this study seeks to explore not only the functional role of AI in primary care but also the emotional, relational, and organizational consequences of its implementation. This comprehensive framework enables a nuanced understanding of how AI can be both a support and a burden in PCPs’ lived experience.

### 1.2. Current State of Research

AI has gained significant attention as a tool to improve diagnostic accuracy, optimize clinical workflows, and address workforce shortages in primary care. Applications such as clinical decision support systems (CDSS), predictive analytics for chronic disease management, and automated documentation tools are already in use or development [27]. However, research paints a complex and ambivalent picture of AI’s actual impact on physicians’ everyday work.

#### 1.2.1. Opportunities in the Use of AI in Primary Care

Many studies highlight AI’s potential benefits. AI can assist in diagnostic reasoning [28,29], triage, and risk stratification [30], often complementing or outperforming human judgment in specific domains. AI is also often credited with reducing administrative burden through automation, potentially freeing time for direct patient care [31]. Physicians often express cautious optimism about AI improving efficiency and supporting complex decisions, especially in resource-constrained environments [32]. Early qualitative data suggest some clinicians view AI as helpful in reducing cognitive load and enhancing guideline adherence [33].

#### 1.2.2. Challenges in Using AI in Primary Care

Despite these advantages, integrating AI into clinical workflows remains challenging. Physicians report that AI tools often disrupt workflows, increase screen time, and poorly align with primary care realities [7]. Concerns about data quality, interpretability, and potential over-reliance on AI recommendations persist [5,34]. Lack of transparency and explainability can reduce trust and increase cognitive workload [8,35]. Additionally, AI raises new ethical and legal uncertainties around responsibility for errors or misclassifications [36,37,38]. Emerging studies indicate AI may contribute to technostress, particularly when perceived as unreliable or burdensome [24]. Physicians may also fear loss of professional identity, de-skilling, depersonalization of care, or diminished clinical roles [23,39].

### 1.3. Study Aim

This qualitative study aims to explore primary care physicians’ experiences, perceptions, and attitudes toward AI use in their clinical practice. Specifically, it investigates whether PCPs view AI as a supportive tool that enhances their work or as an additional source of burden, including its effects on workload, decision-making, and the doctor–patient relationship. Furthermore, the study seeks to identify the resources and conditions that enable successful and sustainable AI integration in primary care.

The main research question is as follows: How do primary care physicians perceive the impact of artificial intelligence on their clinical practice and workload?

The following sub-questions should also be addressed:

1. What types of AI applications are currently used in primary care, and how are they integrated into existing workflows?
2. To what extent do physicians view AI as a tool that supports decision-making versus a source of additional cognitive or administrative burden?
3. How do PCPs experience the influence of AI on doctor-patient interaction and the quality of care?
4. What concerns do physicians have regarding responsibility, safety, and reliability of AI in patient care?
5. What psychological effects, such as stress, anxiety, or relief, do physicians associate with the use of AI tools in everyday practice?
6. What resources or conditions (e.g., training, infrastructure, support systems) are necessary for the successful and stress-reducing implementation of AI in primary care?

## 2. Materials and Methods

### 2.1. Study Design

This study employed a qualitative research design using semi-structured interviews to explore primary care physicians’ experiences, perceptions, and attitudes regarding the use of AI in clinical practice. A qualitative approach was chosen to gain an in-depth understanding of the nuanced ways in which AI is perceived as supportive or burdensome in everyday healthcare contexts. The study followed the COREQ (Consolidated Criteria for Reporting Qualitative Research) guidelines for reporting qualitative research.

### 2.2. Participant Recruitment and Sampling

Participants were recruited through purposive sampling to ensure a diverse range of perspectives. Eligible participants were licensed primary care physicians (including general practitioners and internists) currently practicing in outpatient settings in Germany. Efforts were made to include participants of varying ages and years of professional experience, as well as practice settings (urban vs. rural). Recruitment was conducted via professional networks and personal contacts. A total of n = 28 interviews were conducted, with saturation reached when no new themes emerged.

### 2.3. Data Collection

Data were collected between September and December 2024 using semi-structured interviews based on a pre-developed interview guide. The interview guide included open-ended questions covering themes such as perceptions of AI, its perceived benefits and challenges, impact on workload and the doctor-patient relationship, and required resources for successful implementation. The guide was pilot tested with two physicians and refined accordingly.

Interviews were conducted via video conferencing or in person, depending on participant preference and public health guidelines. Each interview lasted approximately 45 to 60 min. All interviews were conducted in German, recorded with informed consent, and transcribed verbatim. Transcripts were anonymized, and pseudonyms were assigned to protect participant confidentiality.

### 2.4. Data Analysis

The data were analyzed using thematic analysis as described by Clarke and Braun (2006) [40], allowing for the identification of patterns across the dataset. The analysis followed a six-step process: familiarization with the data, generating initial codes, searching for themes, reviewing themes, defining and naming themes, and producing the final report. Coding was conducted using the software MAXQDA (version 24), and an initial codebook was developed inductively from the data.

To enhance reliability, two researchers independently coded a subset of transcripts and discussed discrepancies until consensus was reached. Reflexivity was maintained throughout the analysis by documenting analytic decisions and reflecting on the researchers’ own assumptions and potential biases.

### 2.5. Criteria for Assessing Qualitative Research

We addressed the following quality criteria for our study: 1. Procedural rigour (i.e., consistency, transparency, etc.); 2. Representativeness; 3. Clarification and justification; 4. Interpretative rigour; 5. Reflexivity; and 6. Transferability (see Table 1).

### 2.6. Ethical Considerations

This study involved human participants and was therefore conducted by the Declaration of Helsinki and approved by the Ethics Committee of the University Medical Center Hamburg-Eppendorf, Germany (LPEK-0678). Participants gave informed consent to participate in the study before taking part.

### 2.7. Use of AI Software

In the preparation of this manuscript, AI-based language and literature research tools (specifically ChatGPT https://chatgpt.com/, Elicit, Deepl-Translate) were utilized to assist with the literature search and to improve the clarity and accuracy of the English language. Due to limited personnel resources and the absence of access to professional translation services, these tools supported ensuring linguistic correctness and coherence. All intellectual content, analysis, and interpretation remain the sole responsibility of the authors. The use of AI tools did not influence the scientific integrity or originality of the manuscript.

## 3. Results

### 3.1. Sociodemographic Data

Table 2 provides an overview of the participants’ sociodemographic characteristics, highlighting a majority of general practitioners and a balanced gender distribution. Participants were primarily aged 36 to 45 years, with varied levels of professional experience ranging from 10 to over 35 years. This diverse sample supports a comprehensive exploration of perspectives within the study.

### 3.2. Qualitative Results on AI Use in Primary Care

Table 3 summarizes the key thematic categories identified from qualitative interviews with primary care physicians regarding the use of artificial intelligence (AI). It highlights both the perceived opportunities and benefits as well as the challenges, concerns, and future conditions necessary for successful AI adoption in clinical practice. These themes provide a comprehensive overview of physicians’ experiences and perspectives on AI’s evolving role in primary care. Table S1 (see supplement) presents a comprehensive overview of main thematic categories derived from the interviews. Each theme is illustrated with representative quotes.

### 3.3. Perceptions and Utilization of AI in Primary Care

Participants widely regarded AI as a promising asset that enhances clinical decision-making by rapidly processing complex datasets. Many physicians reported integrating AI tools, such as automated reminders and risk assessments, into their daily routines to support various tasks efficiently. Despite recognizing AI’s potential to improve diagnostic accuracy and preventive care, the consensus strongly emphasized that clinical judgment remains the cornerstone of medical practice. As one general practitioner expressed, “*AI is a valuable adjunct in primary care. It may help us process information faster and flag risks we might otherwise overlook. But at the end of the day, it’s still the clinician who makes the final call.*” (GP, male, 36–45 years).

AI’s role in prevention and early detection was particularly highlighted, with physicians noting its ability to identify high-risk patients for targeted interventions, thus contributing to improved long-term health outcomes.

An internist reflected, “*With AI, we may be able to identify high-risk patients early and focus preventive efforts more precisely. This may ultimately improve our patients’ long-term health*.” (Internist, female, 36–45 years). Additionally, AI’s contribution to personalized medicine was recognized as a major advance, helping tailor treatment plans to individual patient needs. A physician stated, “*AI may help create individualized treatment plans tailored precisely to each patient—this could represent the future of medicine.*” (GP, female, 46–55 years).

Furthermore, physicians valued AI’s capacity to provide up-to-date medical knowledge, keeping them informed amid rapid advancements in medicine. One general practitioner commented, “*Medicine advances so rapidly that we can’t keep up with everything ourselves. AI systems can provide us with the latest studies and guidelines.*” (GP, male, 56–65 years).

AI was also seen as a crucial support for primary care in rural and underserved areas, where specialist access is limited, helping bridge healthcare disparities. As a physician noted, “*AI can support physicians in remote areas where access to specialists is limited. This benefits patients far from major cities.*” (Internist, female, 56–65 years).

Taken together, these perspectives illustrate how AI is integrated as a supportive tool in primary care, augmenting physician capabilities while underscoring the irreplaceable role of human clinical judgment.

### 3.4. AI as Support: Opportunities and Benefits

Several participants emphasized the practical benefits of AI in primary care, particularly its role in alleviating administrative burdens. By automating time-consuming tasks such as documentation and scheduling, AI frees up valuable time that physicians can dedicate to direct patient interaction. As one general practitioner noted, “*AI may help free up time by automating administrative tasks, allowing me to focus more on meaningful interactions with my patients. It may provide valuable support when dealing with complex clinical decisions.*” (GP, female, 36–45 years).

Similarly, another general practitioner described AI tools as “[…] *the biggest relief in administrative work*” (GB, male, 36–45 years), highlighting how these technologies enable more patient-centered care.

Beyond administrative support, AI was valued as a diagnostic aid. Physicians appreciated its ability to detect subtle symptoms and improve diagnostic confidence, ultimately enhancing care quality. A primary care physician reflected, “*AI has become an invaluable assistant, especially when I’m faced with complex cases. It helps me consider diagnoses I might not have thought of, improving patient care.*” (Internist, male, 36–45 years).

Another doctor pointed out that “*The use of AI in preventive screening may enable earlier identification of at-risk patients, potentially transforming long-term health outcomes,*” reinforcing AI’s potential in early detection and prevention (GP, male, 36–45 years).

Overall, these insights illustrate how AI, when used transparently and as a supplementary resource, not only streamlines workflow but also supports clinical decision-making, contributing to improved patient outcomes and strengthening trust in care.

### 3.5. Challenges and Additional Burdens

While AI offers clear benefits in enhancing clinical workflows and patient care, many primary care physicians articulated substantial challenges and burdens associated with its integration. A major concern involves the frequent generation of excessive alerts and irrelevant recommendations, which disrupt daily workflow and create additional stress. Rather than alleviating the administrative and cognitive load, these interruptions sometimes exacerbate physician fatigue. One internist explained, “*Sometimes the AI throws up alerts that don’t make sense in the context of the patient. I spend more time dismissing pop-ups than focusing on care. And when it contradicts my judgment, it creates doubt rather than clarity.*” (Internist, male, 46–55 years).

Another key issue is the lack of transparency in AI decision-making, often described as the “black box” phenomenon. Physicians expressed discomfort with relying on AI outputs without understanding the underlying logic, which challenges trust and acceptance. A primary care physician reflected, “*The biggest challenge with AI is not knowing how it reaches its conclusions. This opacity makes me hesitant to rely on its recommendations fully, especially when my clinical experience suggests otherwise.*” (Internist, male, 36–45 years).

Another noted, “*Sometimes AI recommendations feel like a black box. I worry about relying too much on something I don’t fully understand or control*.” (GP, male, 46–55 years).

Beyond technical limitations, physicians voiced concerns about legal liability. The question of responsibility when AI-guided decisions lead to errors was described as a significant source of anxiety. As one physician remarked, “*There’s also the pressure of legal responsibility. If an AI tool makes a mistake, where does that leave me as the clinician?*” (GP, male, 46–55 years). This uncertainty may deter full adoption or encourage defensive practice behavior.

Data privacy and cybersecurity risks were also frequently mentioned. Given the sensitive nature of patient information, many physicians worried whether AI systems are adequately protected against breaches. For example, an internist commented, “*Patient data is extremely sensitive. I worry whether all AI applications are sufficiently protected against cyberattacks.*” (Internist, female, 46–55 years).

Furthermore, the technological infrastructure in many primary care settings is not yet prepared for smooth AI integration. Compatibility issues with existing practice software and frequent technical glitches contribute to frustration and hinder efficient use. As a physician noted, “*Our practice software is not designed for AI integration. The interplay often doesn’t run smoothly, which is frustrating.*” (GP, male, 36–45 years).

A critical barrier is the lack of sufficient training and educational resources to help clinicians understand and optimally use AI tools. Many physicians reported feeling unprepared and unsupported, stating, “*We hardly have time or opportunities to learn complex systems. Without thorough training, using AI is hard to imagine for me.*” (GP, male, 56–65 years).

Another physician expressed, “*Introducing AI without proper training leaves many of us anxious and unsure.*” (GP, female, 56–65 years).

Concerns about the depersonalization of care emerged repeatedly. Physicians worried that increased reliance on AI might undermine the essential human connection with patients. One family physician said, “*I worry that technology might replace the personal relationship with patients. The trust between doctor and patient is the foundation of our work.*” (GP, female, 46–55 years).

Additional burdens include the risk of increased documentation time and administrative workload. Rather than reducing work, some physicians feared AI might shift their focus away from patient interaction. A general practitioner shared, “*If I have to spend more time on documentation due to AI, there’s less time left for patients. That would be a step backward.*” (GP, male, 55–65 years).

Bias and fairness in AI algorithms were also highlighted as important ethical concerns. Since AI systems are trained on datasets that may not represent all population groups equally, physicians cautioned about the risk of perpetuating disparities in diagnosis and treatment. One physician noted, “*AI systems rely on data that do not always represent all population groups, which can lead to biased diagnoses.*” (GP, male, 46–55 years).

Finally, the high costs associated with purchasing, implementing, and maintaining AI technologies raised questions about economic feasibility, especially in smaller or resource-limited practices. A participating physician expressed this concern succinctly: “*The purchase and ongoing operation of such systems are expensive. I wonder if this really makes sense for a small practice.*” (Internist, male, 56–65 years).

### 3.6. Impact on the Physician–Patient Relationship

Participants expressed mixed views on how AI affects the therapeutic relationship in primary care. Some physicians voiced concerns that increased reliance on AI could weaken the essential personal connection with patients, potentially leading to perceptions of reduced empathy and trust. As one family physician explained, “*There’s a risk that patients might feel we’re more focused on the screen or the algorithm than on them, which could undermine trust and empathy.*” (GP, male, 46–55 years).

Similarly, another family physician shared worries that “*Too much reliance on AI might reduce the personal connection I have with my patients, which is fundamental to good care.*” (GP, female, 36–45 years).

Conversely, others highlighted that transparent communication about AI’s supportive role can strengthen patient confidence. A general practitioner noted, “*When I explain how AI supports my decisions, patients often feel reassured that we’re using the best available tools to provide comprehensive care.*” (GP, female, 36–45 years). This suggests that integrating AI thoughtfully and openly can enhance the therapeutic alliance rather than detract from it.

### 3.7. Responsibility and Safety Concerns

A prominent and recurrent theme among participants was the significant responsibility physicians carry when making clinical decisions supported by AI. While AI can offer valuable insights, many physicians expressed a cautious approach to relying on AI outputs, especially when the underlying decision-making processes remain unclear. This uncertainty heightened their demand for transparency and accountability in AI systems to ensure patient safety and maintain trust. As one primary care physician emphasized, “*Ultimately, the responsibility for patient safety rests with us. We can’t blindly follow AI recommendations without knowing how they’re generated. Transparency is crucial to maintain trust and accountability.*” (GP, female, 46–55 years)

This perspective highlights the delicate balance physicians must maintain between leveraging AI assistance and preserving their professional judgment and ethical obligations.

### 3.8. Psychological Burden and Resources

Physicians frequently reported feelings of uncertainty and anxiety when adapting to new AI technologies, particularly in the absence of adequate guidance and training. This lack of support contributed to psychological strain and hesitancy towards AI adoption. Participants emphasized the critical need for comprehensive education, continuous technical assistance, and meaningful involvement in the AI development process to foster acceptance and confidence. As one general practitioner noted, “*Introducing AI without proper training leaves many of us anxious and unsure. To truly benefit, we need ongoing support and a seat at the table when these tools are developed.*” (Internist, female, 56–65 years)

Similarly, a physician shared, “*The uncertainty around AI recommendations can be quite stressful. Without clear guidance, it sometimes feels like we’re navigating in the dark.*” (GP, female, 36–45 years)

These insights underscore that beyond technological capabilities, the successful integration of AI in primary care depends heavily on addressing the emotional and educational needs of physicians.

### 3.9. Future Perspectives and Conditions for Successful Integration

Many participants expressed optimism about the future role of AI in primary care, viewing it as a valuable tool with great potential to enhance clinical practice. However, they emphasized that the successful adoption of AI critically depends on its design being user-friendly and aligned with the practical, day-to-day needs of physicians. Meaningful involvement of healthcare professionals in the development process was seen as essential to ensure that AI systems function as true assistants rather than additional burdens. Participants also highlighted the importance of organizational support and educational resources to facilitate a smooth transition to AI-enhanced care.

As one general practitioner explained, “*We see great potential in AI, but for it to truly help, it must be easy to use and designed with our input. Otherwise, it just adds another layer of complexity instead of easing our workload*.” (Internist, male, 56–65 years)

Another physician added, “*With thoughtful design and meaningful collaboration, AI could become an indispensable partner in primary care, enhancing our capabilities without overwhelming us.*” (GP, female, 36–45 years)

Echoing this sentiment, a physician reinforced this point: “*For AI to be more than a burden, it must be user-friendly and truly tailored to our daily needs*.” (GP, male, 36–45 years)

These perspectives underline that for AI to be effectively integrated into primary care, it must be developed collaboratively with clinicians and supported by appropriate training and organizational structures.

## 4. Discussion

This qualitative study explored how primary care physicians (PCPs) perceive the integration of artificial intelligence (AI) into their clinical practice, particularly regarding its impact on workload, decision-making, and the doctor–patient relationship. The findings reveal a complex and ambivalent picture: while AI is generally recognized as a supportive tool with the potential to improve efficiency and care quality, it is also associated with new challenges, emotional strain, and systemic demands that may exacerbate workload rather than alleviate it.

### 4.1. AI as a Supportive Tool: Opportunities and Benefits

Consistent with previous research [27,41,42,43], many participants in this study identified tangible benefits of AI, particularly in automating administrative tasks such as documentation and scheduling. These functions were perceived as reducing time pressure and allowing more focus on direct patient care; an effect that has been associated with improved job satisfaction and reduced burnout [44]. Moreover, AI’s analytical capabilities were seen as helpful in supporting diagnostic decision-making, especially in complex cases, which aligns with literature highlighting AI’s potential in augmenting clinical judgment [45].

Interestingly, some physicians reported that transparent and collaborative use of AI could foster patient trust, as it demonstrated thoroughness and use of cutting-edge tools. This finding contrasts with commonly voiced concerns that technology might depersonalize care [46,47], suggesting that the relational effects of AI depend significantly on how it is introduced and communicated within the clinical encounter.

### 4.2. Ambivalence and Burden: Risks of Overload and Cognitive Strain

Despite these advantages, our findings also highlight critical challenges. Several participants experienced AI tools as intrusive or disruptive when poorly integrated into existing workflows. Frequent alerts, irrelevant suggestions, or interface issues contributed to frustration and increased cognitive load, echoing concerns in the literature about “alert fatigue” and “digital burnout” [48,49].

More notably, participants expressed a strong sense of psychological burden arising from a perceived loss of autonomy or increased pressure to align with AI-generated recommendations. This aligns with concerns from other studies about “automation bias” and the erosion of professional confidence when clinicians feel compelled to defer to opaque algorithms [50,51,52,53]. The “black box” nature of many AI systems, combined with a lack of transparency and limited understanding of underlying mechanisms, fueled uncertainty and anxiety among users, particularly in high-stakes decision-making contexts [54].

### 4.3. Impact on the Doctor–Patient Relationship

The study also sheds light on the nuanced effects of AI on the therapeutic relationship. Some physicians feared that AI use could weaken personal interactions and undermine empathy, particularly if patients perceived technology as replacing, rather than supporting, human care. This concern reflects broader ethical debates about depersonalization in technologically mediated healthcare [55,56]. At the same time, others emphasized that AI could enhance trust and communication when used transparently and collaboratively, highlighting the importance of implementation strategies that preserve core values of primary care, such as continuity, empathy, and relational trust [9,57,58].

### 4.4. Accountability, Safety, and Ethical Concerns

A dominant concern across interviews was the issue of accountability: if AI tools contribute to clinical decisions, who is ultimately responsible for their consequences? Physicians consistently emphasized that legal and ethical responsibility remains with the human clinician, even when decisions are algorithmically informed. This reinforces calls from previous research for clearer regulatory frameworks, robust validation processes, and mechanisms for explainability and auditability in AI applications [9,59,60]. Without these safeguards, physicians may remain hesitant to fully embrace AI in critical aspects of care.

### 4.5. Preconditions for Sustainable Implementation

Participants highlighted several essential conditions for successful, stress-reducing AI integration: user-friendly design, adequate training, technical support, and active involvement of clinicians in the development and evaluation of AI tools. These factors are consistent with the implementation of science frameworks emphasizing stakeholder engagement, contextual fit, and capacity-building as prerequisites for sustainable technology adoption [23,61].

Our findings indicate that digital literacy and professional experience significantly shape physicians’ attitudes toward artificial intelligence. Younger, more digitally proficient participants generally expressed greater openness to AI applications, whereas more experienced physicians tended to be more cautious or skeptical. This aligns with previous research: Pinto dos Santos et al. (2019) found that medical students with higher digital literacy held more positive views of AI compared to older practitioners [62]. At the same time, Naik et al. (2022) highlighted that experienced physicians often express concerns regarding reliability, liability, and ethical issues, which may explain their skepticism [63]. In this context, Misra et al. (2024) emphasized the need to tailor training and educational programs to accommodate varying levels of familiarity and comfort with technology in order to foster acceptance and effective use of AI [64]. These insights should inform the development of future implementation strategies to optimally support both younger and more experienced clinicians.

### 4.6. Strengths and Limitations

This study offers several notable strengths that enhance the validity and relevance of its findings. First, it provides rich, qualitative insights into the lived experiences of primary care physicians, a group whose perspectives are often underrepresented in AI research. By employing in-depth, semi-structured interviews and reflexive thematic analysis, the study captures the nuanced, context-sensitive perceptions that may not emerge through quantitative methods alone.

Second, the study is among the first to explore the real-world implications of AI adoption in routine primary care from a human-centered perspective. This approach advances the current literature, which often focuses predominantly on technical performance, by highlighting the social, ethical, and psychological dimensions of implementation.

Third, the study was conducted during a period of growing, yet uneven, digital transformation in healthcare. This timing provides a unique window into the early-stage challenges, expectations, and ambivalences surrounding AI technologies, insights that are especially valuable for informing future implementation strategies.

However, this study has several limitations that warrant consideration. First, the sample size was relatively small and limited to German primary care settings, which may constrain the generalisability of the findings to other healthcare systems. Second, the potential for self-selection bias must be acknowledged, as physicians with stronger pre-existing opinions or interest in AI may have been more inclined to participate. Third, while thematic analysis enabled an in-depth exploration of subjective experiences, it remains inherently interpretative and influenced by the researchers’ analytic lens.

Moreover, participants referred to diverse conceptualisations of AI based on their individual encounters, and no single AI system was assessed in a standardized manner. The study also focused exclusively on the perspectives of general practitioners, omitting the views of other key stakeholders such as medical assistants, IT personnel, or patients. Finally, the cross-sectional design offers a temporal snapshot; attitudes and practices may evolve as AI systems become more widespread, and clinicians gain practical experience. Future research could benefit from mixed-method approaches, integrating observational data and quantitative metrics, such as workload, decision accuracy, and clinician wellbeing—to complement these qualitative insights.

### 4.7. Research Implications

This study contributes to the expanding body of scholarship on the integration of artificial intelligence (AI) in primary care by providing nuanced, qualitative insights into the lived experiences of practising physicians. The findings highlight the ambivalent and often paradoxical perceptions surrounding AI, positioning it simultaneously as a valuable clinical support tool and a potential source of disruption within established professional routines.

These results substantiate the core assumptions of socio-technical systems theory, which frames this study, by illustrating the critical importance of alignment between social and technical subsystems in healthcare settings. Specifically, experiences of workflow disruption, increased cognitive and emotional burden, and concerns about loss of professional autonomy exemplify the real-world consequences of socio-technical misalignment. Conversely, positive experiences related to enhanced diagnostic support and improved preventive care underscore the potential benefits when such alignment is achieved.

Beyond confirming existing theory, our findings extend socio-technical perspectives by illuminating the psychological, relational, and ethical dimensions of AI implementation, areas that have been less explicitly addressed in prior literature. This suggests a need to broaden the conceptualization of alignment to explicitly incorporate these affective and moral factors, which significantly influence technology acceptance and clinical practice.

Consequently, future theoretical and empirical work should further explore several key concepts emerging from our study, including the following:

- The dynamics of trust and transparency in clinician–AI interactions
- The negotiation and preservation of professional autonomy in the context of algorithmic recommendations
- The evolving frameworks of accountability and responsibility when AI supports clinical decisions
- The role of digital literacy and organizational infrastructure in facilitating sustainable socio-technical integration.

A more comprehensive socio-technical framework that integrates these dimensions will be crucial for understanding and guiding the ethical, effective, and context-sensitive adoption of AI in primary care.

In addition, longitudinal studies are essential to elucidate the sustained effects of AI implementation on key indicators such as physician workload, diagnostic accuracy, clinical decision-making, job satisfaction, and patient-centered outcomes. Such studies would enable a more precise understanding of the cumulative and potentially delayed consequences of AI integration over time.

Finally, to advance a more generalisable and context-sensitive understanding of AI adoption, comparative research across diverse healthcare systems, organizational cultures, and national contexts is warranted. Investigating the sociotechnical dynamics that facilitate or hinder AI acceptance in varied settings will yield critical insights into the conditions under which AI can be responsibly and effectively embedded in routine clinical care.

### 4.8. Practical Implications

For artificial intelligence (AI) to realise its full potential in primary care, its implementation must transcend mere technical deployment and actively engage with the broader organizational, relational, and ethical contexts in which it operates. Based on the findings of this study, several key practical considerations emerge as outlined.

#### 4.8.1. System Design and Integration

Effective AI systems should be intuitive, user-centered, and seamlessly integrated into existing clinical workflows. Moreover, transparency in algorithmic reasoning is essential to foster clinician trust, enhance interpretability, and minimize cognitive load during decision-making processes.

#### 4.8.2. Training and Digital Literacy

Tailored training programmes are crucial to accommodate varying levels of digital literacy among physicians. Such training must not only address technical competencies but also equip users with a critical understanding of the ethical and legal dimensions of AI-assisted care. In addition, it is essential that foundational education on AI and its implications for clinical practice be embedded into the undergraduate curricula of all health professions.

#### 4.8.3. Organizational Infrastructure and Change Management

Successful AI implementation requires adequate institutional support, including robust IT infrastructure, time allocations for system adaptation, and ongoing technical assistance. Organizational leadership plays a pivotal role in actively managing the cultural and procedural transitions associated with digital innovation.

#### 4.8.4. Preserving the Human Dimension of Care

AI should be designed and implemented to augment, rather than replace, the human aspects of clinical practice. Transparent communication with patients regarding the role of AI in their care can help preserve and potentially strengthen the therapeutic alliance.

#### 4.8.5. Ethical and Regulatory Frameworks

Clear legal and ethical guidelines are essential to ensure responsible use. Regulatory authorities must provide comprehensive frameworks that address liability, data privacy, algorithmic accountability, and the boundaries of autonomous decision-making in clinical settings.

By systematically addressing these practical, ethical, and organizational dimensions, AI can evolve into a valuable and sustainable component of primary care, one that supports clinical excellence while safeguarding the foundational values of the medical profession.

## 5. Conclusions

The advent of artificial intelligence has precipitated a paradigm shift within the domain of primary care, albeit one that is intricate in nature. It is neither a universal remedy nor an inherent risk. The ultimate impact of such technology is contingent upon meticulous design, context-sensitive implementation, and judicious integration within extant clinical workflows. Despite the potential of AI to enhance diagnostic accuracy, facilitate personalised preventive strategies and reduce administrative tasks, the findings reveal significant cognitive, ethical, and relational challenges that must be given due consideration. The cautious attitudes adopted by physicians towards AI serve to highlight the necessity for transparency in algorithmic processes and accountability in clinical decision-making. Moreover, concerns regarding workflow disruption and psychological burden underscore the critical need for comprehensive training, continuous technical support, and the meaningful involvement of end users throughout the development of AI. In order to realise the full potential of AI in augmenting primary care without exacerbating clinician workload or undermining the therapeutic relationship, healthcare systems must prioritize collaborative co-design approaches, foster algorithmic explainability, and establish robust organizational infrastructures. It is vital to ensure that AI tools function as intuitive, reliable, and ethically sound assistants if their sustainable adoption is to be achieved, as well as to safeguard the core values of patient-centered care.

## Figures and Tables

**Table 1:** Measures to ensure qualitative research quality.

Quality Criterion	Operationalization	Illustrative Techniques or Procedures
Credibility/Trustworthiness	Ensure that the findings accurately capture participants’ viewpoints without undue influence or distortion.	Validation through participant feedback loops (e.g., respondent validation).
Dependability/Consistency/ Reliability	Maintain transparency and traceability throughout the research process.	Documented decision pathways and use of consistent data handling protocols (e.g., methodological logbook).
Clarification Accuracy	Ensure accurate understanding of participants’ statements and reduce ambiguity.	Use of probing and clarification questions; restating participants’ responses for confirmation.
Justification/Reasoning Transparency	Explore underlying motives, rationales, or interpretive frameworks behind participants’ actions or decisions.	Encouraging participants to articulate rationale behind behaviors or viewpoints (e.g., ‘why’ questions).
Objectivity/Confirmability	Demonstrate that interpretations derive directly from the data and are not shaped by researcher bias.	Maintenance of reflective logs and cross-checks with co-researchers to ensure neutrality.
Authenticity/Genuineness	Ensure that participants’ voices are presented faithfully and without distortion.	Application of inductive coding practices; effort to preserve original phrasing and intent.
Reflexivity/Reflective Awareness	Acknowledge and critically examine the researcher’s positionality and potential influence on the research.	Routine documentation of personal assumptions and theoretical lenses (e.g., reflective journaling).
Transferability/Contextual Applicability	Provide sufficient detail to support transfer of findings to other settings or populations.	Thick description of participant characteristics and research context to facilitate relevance assessment.

**Table 2:** Sociodemographic characteristics of the study participants.

Category	Subcategory	n	(%)
**Profession**			
	General Practitioner	20	71.4
	Internist	8	28.6
**Gender**			
	Female	13	46.4
	Male	15	53.6
	Diverse	-	-
**Age Group** **(in years)**			
	36–45	14	50.0
	46–55	8	28.6
	56–65	6	21.4
**Work Experience** **(in years)**			
	10–15	10	35.7
	16–25	9	32.1
	26–35	7	25
	36–45	2	7.2

**Table 3:** Main themes from interviews on AI integration in primary care.

Main Category	Description	Key Themes
Perceptions and Utilization of AI	How AI is perceived and used in everyday primary care practice	Support for clinical decision-making, integration into routine tasks (e.g., reminders, risk assessments), clinical judgment remains central
AI as Support: Opportunities and Benefits	The supportive role of AI in reducing workload and improving care quality	Automation of administrative tasks, improved diagnostic confidence, strengthening patient trust through transparent use
Challenges and Additional Burdens	Challenges and potential burdens associated with AI	Alert fatigue from irrelevant notifications, pressure to conform to AI suggestions, “black-box” issues, uncertainty, and stress
Impact on the Physician–Patient Relationship	AI’s influence on the therapeutic relationship	Concerns about loss of personal connection vs. increased patient confidence through transparent AI integration
Responsibility and Safety Concerns	Physician responsibility and safety considerations when using AI	Caution in following AI recommendations, demand for transparency and accountability
Psychological Burden and Resources	Psychological stress and necessary support resources	Anxiety and uncertainty due to lack of training, need for technical support and involvement in AI development
Future Perspectives and Conditions for Successful Integration	Outlook and prerequisites for successful AI adoption	Optimism about user-friendly design, physician involvement, organizational and educational support for integration

## Data Availability

The datasets analyzed during the current study are not publicly available due to German national data protection regulations but are available from the corresponding author on reasonable request.

## Supplementary Materials

The following supporting information can be downloaded at: https://www.mdpi.com/article/10.3390/clinpract15080138/s1. Table S1: Physicians’ Perspectives on AI in Primary Care: Key Themes and Illustrative Quotes.
