# Supplementary material for: Artificial Intelligence in Primary Care: Support or Additional Burden on Physicians’ Healthcare Work?—A Qualitative Study
Source: Clin Pract. 2025 Jul 25;15(8):138. doi: 10.3390/clinpract15080138 (PMC12384864; doi:10.3390/clinpract15080138)
Supplement: Supplementary file 1 [file clinpract-15-00138-s001.zip › clinpract-3769156-supplementary.pdf]

Table S1

Physicians’ Perspectives on AI in Primary Care: Key Themes and Illustrative Quotes

Main Category	Representative Quotes
**1. Perceptions and Utilization of AI**	“AI is a valuable adjunct in primary care. It helps us process information faster and flag risks we might otherwise overlook. But at the end of the day, it's still the clinician who makes the final call.” — General practitioner, male, 36-45
	“With AI, we may be able to identify high-risk patients early and focus preventive efforts more precisely. This may ultimately improve our patients’ long-term health. — Internist, female, 36-45
	“AI may help create individualized treatment plans tailored precisely to each patient — this could represent the future of medicine.“— General practitioner, female, 46-55
	“Medicine advances so rapidly that we can’t keep up with everything ourselves. AI systems can provide us with the latest studies and guidelines.” — General practitioner, male, 56-65
	“AI can support physicians in remote areas where access to specialists is limited. This benefits patients far from major cities.” — Internist, female, 56-65
**2. AI as Support: Opportunities and Benefits**	“AI may help free up time by automating administrative tasks, allowing me to focus more on meaningful interactions with my patients. It may provides valuable support when dealing with complex clinical decisions.“— General practitioner, female, 36-45
	“AI has become an invaluable assistant, especially when I’m faced with complex cases. It helps me consider diagnoses I might not have thought of, improving patient care.” — Internist, male, 36-45
	“The biggest relief is in administrative work. AI tools that automate documentation and scheduling free up precious time I can spend with my patients.” — General practitioner, male, 36-45

Table S1

Physicians’ Perspectives on AI in Primary Care: Key Themes and Illustrative Quotes

Main Category	Representative Quotes
	“The use of AI in preventive screening may enable earlier identification of at-risk patients, potentially transforming long-term health outcomes.”— General practitioner, male, 36-45
	“AI helps me catch subtle symptoms that might otherwise be overlooked, which gives me more confidence in my diagnoses.” — General practitioner, male, 36-45
**3. Challenges and Additional Burdens**	“Sometimes the AI throws up alerts that don’t make sense in the context of the patient. I spend more time dismissing pop-ups than focusing on care. And when it contradicts my judgment, it creates doubt rather than clarity.” — Internist, male, 46-55
	“The biggest challenge with AI is not knowing how it reaches its conclusions. This opacity makes me hesitant to rely on its recommendations fully, especially when my clinical experience suggests otherwise.” — Internist, male, 36-45
	“Sometimes AI recommendations feel like a black box. I worry about relying too much on something I don’t fully understand or control.” — General practitioner, male, 46-55
	“The technology can be frustrating when it generates false alarms or irrelevant suggestions. It actually adds to my workload rather than reduces it.” — General practitioner, 46-55
	“There’s also the pressure of legal responsibility. If an AI tool makes a mistake, where does that leave me as the clinician?” — General practitioner, male, 46-55
	“I often feel that AI algorithms are impressive, but I can’t always understand how they arrive at their recommendations. That makes me cautious.” — General practitioner, female, 36-45
	“If AI makes a wrong diagnosis, who is responsible? I don’t want to be liable for errors made by a machine.” — Internist, male, 45-56

Table S1

Physicians’ Perspectives on AI in Primary Care: Key Themes and Illustrative Quotes

**Main Category**	**Representative Quotes**
	“Patient data is extremely sensitive. I worry whether all AI applications are sufficiently protected against cyberattacks.” — Internist, female, 46-55
	“Our practice software isn’t designed for AI integration. The interplay often doesn’t run smoothly, which is frustrating.” — General practitioner, male, 36-45
	“We hardly have time or opportunities to learn complex systems. Without thorough training, using AI is hard to imagine.” — General practitioner, male, 56-65
	“Introducing AI without proper training leaves many of us anxious and unsure.” — General practitioner, female, 56-65
	“I worry that technology might replace the personal relationship with patients. The trust between doctor and patient is the foundation of our work.” — General practitioner, female, 46-55
	“If I have to spend more time on documentation due to AI, there’s less time left for patients. That would be a step backward.” — General practitioner, male, 55-65
	“AI systems rely on data that do not always represent all population groups, which can lead to biased diagnoses.” — General practitioner, male, 46-55
	“The purchase and ongoing operation of such systems are expensive. I wonder if this really makes sense for a small practice.” — Internist, male, 56-65
**4. Impact on the Physician-Patient Relationship**	“There’s a risk that patients might feel we’re more focused on the screen or the algorithm than on them, which could undermine trust and empathy.” — General practitioner, male, 46-55.
	“I’m concerned that too much reliance on AI might reduce the personal connection I have with my patients, which is fundamental to good care.” — General practitioner, female, 36-45 years

Table S1

Physicians' Perspectives on AI in Primary Care: Key Themes and Illustrative Quotes

Main Category	Representative Quotes
	"When I explain how AI supports my decisions, patients often feel reassured that we're using the best available tools to provide comprehensive care." — General practitioner, female, 36-45 years<br><br>"I worry that patients may feel we rely too much on machines and lose the human touch that is essential in primary care." — General practitioner, male, 46-55<br><br>"When AI supports decision-making, it can actually enhance trust because patients see that we use every available resource to provide the best care." — General practitioner, male, 36-45
**5. Responsibility and Safety Concerns**	"Ultimately, the responsibility for patient safety rests with us. We can't blindly follow AI recommendations without knowing how they're generated — transparency is crucial to maintain trust and accountability." — General practitioner, female, 46-55)<br><br>"There's also the pressure of legal responsibility. If an AI tool makes a mistake, where does that leave me as the clinician?" — Internist, male, 56-65<br><br>"If AI makes a mistake, I'm still the one held accountable. That's a heavy responsibility to carry." —, Internist, male, 56-65<br><br>"I'm cautious about relying on AI until I understand how it arrives at its conclusions." — General practitioner, female, 36- 45
**6. Psychological Burden and Resources**	"Introducing AI without proper training leaves many of us anxious and unsure. To truly benefit, we need ongoing support and a seat at the table when these tools are developed." — Internist, female, 56-65 years<br><br>"The uncertainty around AI recommendations can be quite stressful. Without clear guidance, it sometimes feels like we're navigating in the dark." — General practitioner, female, 36-45

Table S1

Physicians’ Perspectives on AI in Primary Care: Key Themes and Illustrative Quotes

Main Category	Representative Quotes
	“Using AI sometimes feels like navigating unknown territory without a map. Proper training would help reduce my anxiety.” — General practitioner, female, 36- 45
	“Technical support and ongoing education are essential if AI is going to be a real help instead of a headache.” — General practitioner, male, 46-55
**7. Future Perspectives and Conditions for Successful Integration**	“We see great potential in AI, but for it to truly help, it must be easy to use and designed with our input. Otherwise, it just adds another layer of complexity instead of easing our workload.” — Internist, male, 56-65
	“With thoughtful design and meaningful collaboration, AI could become an indispensable partner in primary care—enhancing our capabilities without overwhelming us.” — General practitioner, female, 36-45
	“I believe AI will become an indispensable part of primary care, but only if it’s designed with input from physicians.” — General practitioner, male, 36-45
	“For AI to be more than a burden, it must be user-friendly and truly tailored to our daily needs.” — General practitioner, male, 36-45)
**8. Mixed / Reflective Views**	“AI is a tool, not a replacement. It’s helpful when used wisely, but it’s important we don’t lose sight of our clinical judgment and patient relationships.” — General practitioner, female, 46-55
	“The potential is huge, but we need proper training and support. Otherwise, the technology risks becoming just another source of stress.” — General practitioner, male, 36-45
